# Quantitative research into the deconditioning of hemodynamic to disorder of consciousness carried out using transcranial Doppler ultrasonography and photoplethysmography obtained via finger-transmissive absorption

**DOI:** 10.1007/s10072-015-2429-1

**Published:** 2016-01-12

**Authors:** Zhen Liu, Yan Zhou, Rui Yi, Jianghong He, Yi Yang, Li Luo, Yiwu Dai, Xiaomin Luo

**Affiliations:** Department of Neurosurgery, No. 263 Clinical Department of Beijing Army General Hospital, Beijing, 101149 China; Department of Internal Medicine, TongZhou Maternal and Child Health Hospital of Beijing, Beijing, 100000 China; Department of Neurosurgery, Beijing Army General Hospital, Beijing, 100700 China; Healthcare Department, Beijing Genomics Institute, Shenzhen, 518083 China

**Keywords:** Disorder of consciousness, Photoplethysmography, Transcranial Doppler ultrasonography, Coma Recovery Score-Revised, The deconditioning of the cardiovascular system

## Abstract

In this study, transcranial Doppler ultrasonography (TCD) and photoplethysmography (PPG) have been utilized, through the observation of peripheral and cerebrovascular hemodynamic changes of the disorder of consciousness (DOC) patients, measured on clinical behavior scale of Coma Recovery Score-Revised (CRS-R) to obesrve their diagnostic value in evaluation of DOC patients. TCD ultrasound was used to evaluate the flow velocity and waveform patterns of middle cerebral artery (MCA), while PPG infrared signals were utilized to assess the peripheral circulation as a mean of measuring cardiovascular activities. The research was carried out on a sample of 36 individuals, of which 16 met the DOC criteria and 20 were healthy individuals. Each person in the patients groups was assessed by the CRS-R. The velocity of middle cerebral artery in tested patients in a whole cardiac cycle, detected by TCD, decreased comparing with normal values. The values of pulsatility index (PI) of the MCA increased in patients groups comparing with normal. Through binary variables correlation analysis, we found that the PI of the left MCA of TCD of the patients significantly inversely correlated with their motor subscore, included in their CRS-R in the level of *α* = 0.05 (Pearson’s product-moment correlation coefficient = −0.556, *p* = 0.025). The values of photoplethysmographic augmentation index (PAI) that were detected by PPG increased comparing with normal. Finally, using binary variables correlation analysis we found the significant inverse correlation between the PAI of PPG and the mean velocity of the left MCA of the TCD in the level of *α* = 0.05 (Pearson’s product-moment correlation coefficient = −0.377, *p* = 0.022) in all the groups. The results of this study revealed a specific relationship between PI and PAI in the DOC patients. That relationship can potentially be exploited to enhance the capabilities in early assessment of the deconditioning of the DOC patients’ cardiovascular system and its influence on their cerebral vascular system. Ultimately, the dependency discovered can assist in predicting the tendency of the prognosis of the DOC patients in clinic.

## Introduction

Patients with disorder of consciousness (DOC) are individuals, who following coma recovery often seem awake but unaware, unresponsive or low-responsive to their environment. While some of them are capable of recovery and do eventually regain minimal responsiveness to external stimuli (known as “minimally conscious state”), which can ultimately lead to full recovery of consciousness and responsiveness, many are unable to recover and maintain their low-responsive, living on in the persistent vegetative state (PVS) [1].

In the early stages of the condition, assessment of patients’ recovery chance with DOC is a critical issue. Through MRI and PET–CT, a number of researchers have found that cerebral blood flow (CBF) and brain metabolism of the patients with DOC are significantly decreased, and that these changes correlate with the development of the condition [2]. In other studies on arterial spin labeling (ASL); which is a magnetic resonance perfusion method was used to measure the differences of CBF between patients with DOC and normal controls, was used. A general decrease of CBF was found among tested patients in these studies, whilst a longitude observation of one subject with increase of global CBF values showed clinical improvement [3],suggesting that the change of brain blood stream dynamics in patients with DOC may provide some indicators of probability of the patients’ recovery.

While these methods provide higher diagnostic value, their clinical application is limited due to their relative complexity and cost. Fortunately, transcranial Doppler ultrasonography (TCD), a kind of low-cost and practical diagnostic method, has the ability to indirectly show brain’s cerebral perfusion and metabolic state via measurements of cerebral artery blood flow velocity, spectrum parameters, shape of hemodynamic changes and the spectromorphology of the blood flow [4, 5]. However, even if it might allow good diagnostic value for DOC patients, providing accurate diagnosis in DOC cases would be difficult due to the limitation of TCD technology.

Moreover, the deconditioning of cardiovascular system due to immobility among DOC patients is also a well-known problem which influences the underlying illness in a negative way [6–8]. Brain vascular, being the downstream vascular of the cardiovascular system, has been proven to have some impact on the brain blood stream dynamics [9, 10], Being the downstream vascular of the cardiovascular system, brain vascular is influenced by the cardiovascular deterioration, as it is the case in DOC patients, which can negatively affect their underlying condition [6–8]. Thus, the impact of the deconditioning of cardiovascular system on cerebrovascular blood flow dynamics of the DOC patients needs to be evaluated.

Photoplethysmography (PPG) is an optical measurement technique which be used in the microvascular bed of tissue, wave reflections and arterial stiffness can be measured noninvasively using PPG signals which reflect the changes in blood volume with each heart beat, because arterial pulsations are the most significant portion of PPG. PPG’s oscillating component provides a pulsatile wave, whose contour may include content descriptive of vascular health [11]. It is possible to assess, through pulse contour variations, the cardiovascular activities, including wave reflections and arterial stiffness, as the measure of profound alterations in the cardiovascular system of the DOC patients.

In this study, through analyzing the statistical correlations between the hemodynamic parameters which were detected by TCD and PPG technique and CRS-R scale of the DOC patients, we aimed to analyze quantitatively the relationship between the CBF and the cardiovascular deconditioning in DOC patients in combination with their state of consciousness. We hypothesize that a combination of the preceding clinical physiological data will help to improve the understanding of the clinical state and therefore complement the objective indicators of probability of patients’ recovery.

## Method

### Ethics statement

This study has been approved by the Ethics Committee of the Beijing Army General Hospital. Written consent has been obtained from each participant or their guardians.

### Sample selection and grouping

The total sample of the study has been divided into two groups—the patient group and the healthy control group. The patient group analyzed in the study, all of which has satisfied the MCS, or VS criteria [12], has been located either through self-referral, or through inpatients of the Neurosurgery Ward of the Beijing Army General Hospital, between January 2013 and June 2014. All of the subjects in the group were between 22 and 69 years of age, have non-progressive severe brain injury and were in the VS or MCS for no less than 4 weeks at the time of selection, and had no chronic system diseases, such as hypertension, diabetes, hyperlipidemia, etc. Furthermore, subjects who had history of cardiopulmonary arrest during the study, intercurrent infections, a repeated refractory seizure or had used mechanical ventilation were excluded. The final the patient sample consisted of 5 MCS and 11 VS patients, 12 of who were male, four female with mean age of 49.5 ± 18.2 years.

The healthy control group consisted on 20 age-matched individuals, 13 of which who were male, seven female with mean age of 47.7 ± 17.6 years. Subjects in the control group had no history of neurological or systemic illness, head injury, and drug or alcohol abuse (intake).

Additional relevant medical and demographic characteristic of the sample can be seen in Table 1.

**Table 1:** Characteristics and Coma Recovery Scale-Revised (CRS-R) scores of the patients

Patients ID	1	2	3	4	5	6	7	8	9	10	11	12	13	14	15	16
Clinical features																
Sex (age, years)	F66	M66	F28	M57	M50	M40	M53	F69	M22	M56	M49	M23	F49	M63	M42	M56
Etiology	TBI	TBI	ABI	ABI	ABI	ABI	CVA	ABI	TBI	TBI	ABI	TBI	TBI	TBI	CVA	TBI
Months after insult	2	9	2	10	5	6	2	5	36	4	6	10	20	5	24	7
Clinical diagnosis	VS	VS	VS	VS	VS	VS	VS	VS	VS	VS	VS	MCS	MCS	MCS	MCS	MCS
CRS-R																
Auditory function	1	1	1	1	1	1	0	1	1	1	2	1	4	2	2	1
Visual function	0	0	0	0	0	0	0	0	0	1	1	3	1	3	3	3
Motor function	2	2	2	2	2	2	2	3	2	2	1	2	3	3	2	4
Verbal function	1	1	1	1	1	0	1	1	1	1	1	1	3	1	1	1
Communication	0	0	0	0	0	0	0	0	0	0	0	0	2	0	0	1
Arousal function	1	2	2	2	2	2	1	2	2	1	2	2	3	2	2	2
Total function	5	6	6	6	6	5	4	7	6	6	7	9	16	11	10	12

Auditory function: 0—none, 1—auditory startle, 2—localization to sound, 4—consistent movement to command; visual function: 0—none, 1—visual startle, 3—visual pursuit, 5—object recognition; motor function: 0—none, 1—abnormal posturing, 2—flexion withdrawal, 3—localization to noxious; verbal function: 0—none, 1—oral reflexive movement; Communication, 0—none; arousal: 0—none, 1—eye opening with stimulation, 2—eye opening w/o stimulation

*CVA* cerebrovascular accident, *F* female, *M* male, *MCS* minimally conscious state, *TBI* traumatic brain injury, *VS* vegetative state, *ABI* anoxic brain injury

### Clinical evaluation using the Coma Recovery Scale-Revised (CRS-R)

The patients with DOC were assessed by experienced raters using the CRS-R tool. DOC patients’ characteristics and CRS-R are shown in Table 1, inclusive of sensory and cognitive capacities, such as auditory, visual, verbal and motor functions (as well as communication and arousal level), with the total score rating ranging from ‘0’ (worst) to ‘23’ (best).

### TCD methods

Patients’ data for Transcranial Doppler analysis was collected by a single experienced TCD physician at their bedside, with use of an Embo-Dop DWL TCD machine/device, with a 2-MHz pulse-wave Doppler transducer. In the study we used a temporal bone acoustic window, deemed more suitable to assess bilateral middle cerebral artery via cerebral vessel insonation. Artery peak-systolic, end-diastolic velocities and resistive indices were being measured.

### PPG methods

In this study, PPG signals were measured through HC2180-D research platform—an enhanced tool for monitoring waveform analysis development on the basis of HC2180 PPG Blood Flow Monitor, a non-traumatic blood flow parameter detector, released in 2004 by Comperson Biotechnology Co., Ltd. Beijing. PPG, developed in 1937 and much improved since, is a non-invasive method of monitoring blood volume pulsations through detection and temporal analysis of tissue-scattered (absorbed) radiation. With development in microelectronics and computer technologies PPG was introduced into clinical application and is nowadays used as a simplified measure of cardiovascular properties and hemodynamics alterations. As per the measurement process, during the procedure the subjects lay on their backs with both of their upper extremities situated bilaterally parallel to their bodies, with right hand held at the heart level. After 5 min in the resting position, subjects PPG signals start to be recorded via a device on their right index finger, while they stay rested and breathe normally. One day prior to the measurement tested subjects are to refrain from caffeine and drugs of or related to cardiovascular system.

### Statistical method

Acquired data have been expressed as mean ± SD, and the comparison of the parameters of TCD and PPG between the patients and healthy control groups was carried out using an independent sample *t* test. Secondly, comparison between parameters of TCD and CRS-R, PPG and CRS-R, and PPG and TCD was conducted using multiple linear correlation analysis, which was also used to compare PPG with CRS-R score. The statistical analysis was carried out using the SPSS 19.0 software.

## Results

**Fig. 1:**
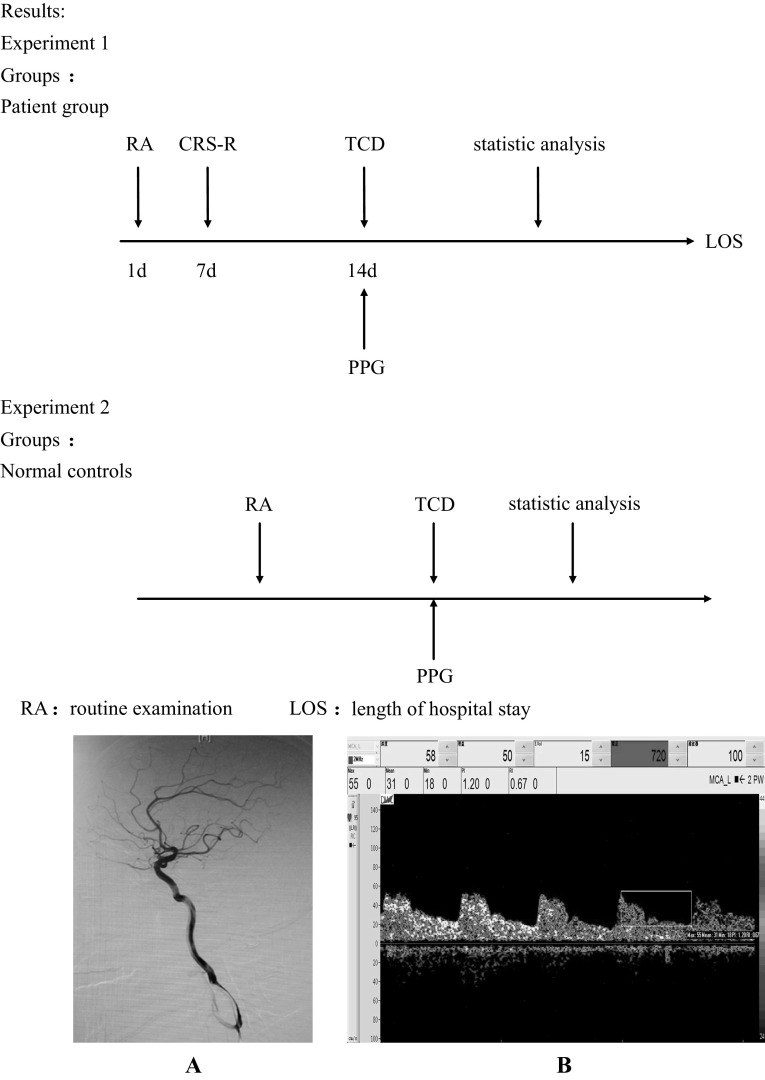
Angiography and TCD of the patient No. 6. **a** Angiography of patient 6 testify normal intracranial artery dissection and keep cerebral circulation. **b** The spectromorphology of the blood flow of left middle cerebral artery which was detected by TCD shows decreased systolic velocity and the end-diastolic velocity

**Fig. 2:**
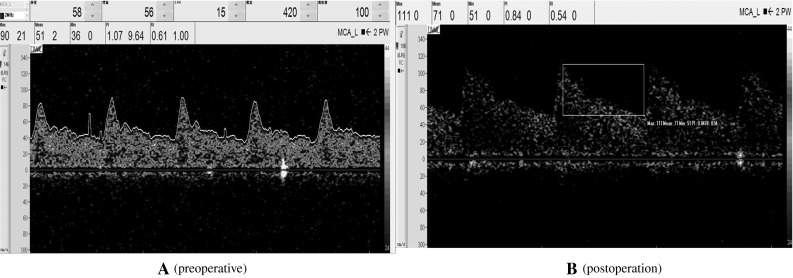
The spectromorphology of the blood flow of left middle cerebral artery which was detected by TCD of the patient No. 13. The patient recovered some of her consciousness and turned MCS from VS state following spinal cord stimulation (SCS). **a** Shows decreased systolic velocity and the end-diastolic velocity before our treatment. But the waveform is relatively normal. The PI is higher than the normal level. **b** Shows after her treatment, the systolic velocity and the end-diastolic velocity is higher than **a** and her PI is decreased in the normal level

Figure 1 shows the angiography of the patient No. 6—artery anatomy within normal and preserved intracranial circulation, and TCD of the left MCA displaying relatively normal waveform pattern, but revealing a decreased systolic diastolic flow and increasing PI. Figure 2 shows the spectromorphology of the blood flow of left middle cerebral artery whi ch was detected by TCD of the patient No. 13—again a normal waveform pattern. It has been noted here, that the patient recovered some of her consciousness and turned MCS from VS state following spinal cord stimulation (SCS). Her TCD revealed increased systolic, diastolic flow and decreased PI in comparison with her past TCD results. Figure 2a, b shows that.

**Fig. 3:**
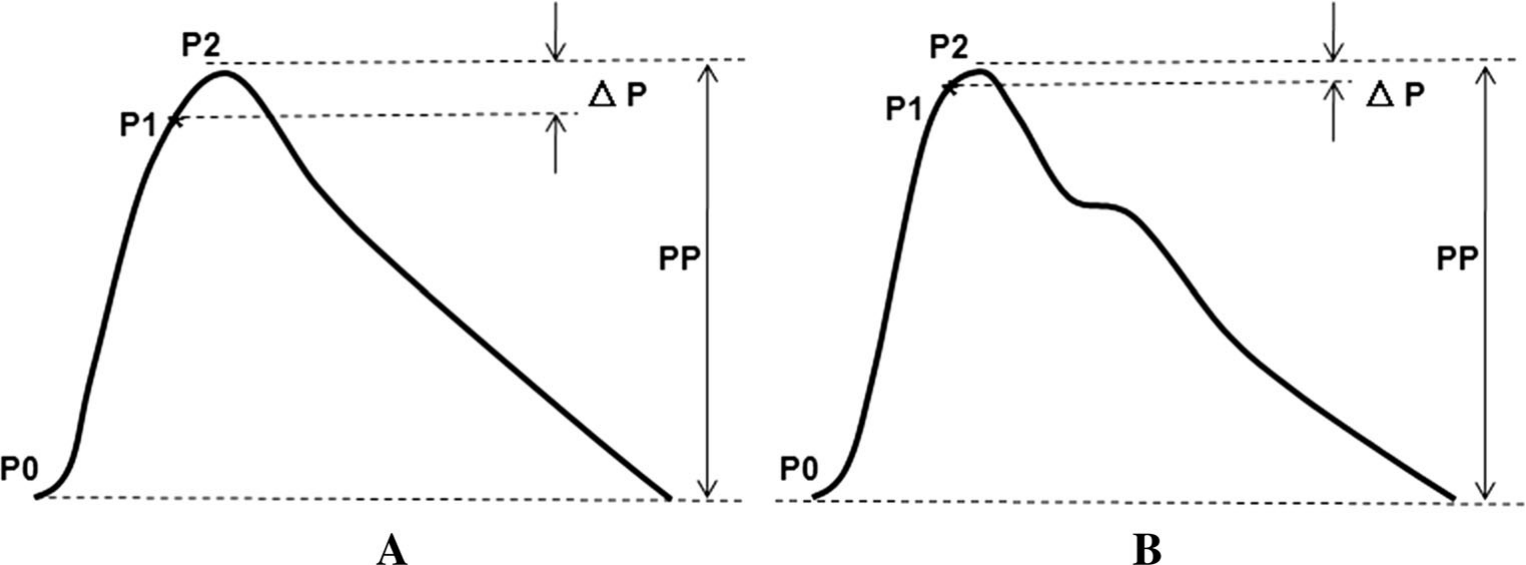
The normalized PPG signal and its feature point used in calculation of PAI for waveforms exhibiting. **a** A DOC patient, **b** a normal control

In our study, the spectromorphology of the blood flow of middle cerebral artery of other patients was similar with Fig. 1b or Fig. 2a. And their waveform patterns were relatively normal. In Fig. 2 we could see that the variations of state of consciousness of the DOC patients could affect their parameters of the CBF but not the waveform patterns of their CBF. Hence, the parameters of the CBF of the left MCA of our study are shown in Table 2—systolic velocity, end-diastolic velocity and mean velocity of the left middle cerebral artery patients, as measured by TCD, were at 75.56, 23.32 and 38.99 cm/s, respectively, amounting to a 23, 43 and 38 % decrease in comparison with normal values. The mean values of the PI of the left MCA increased in patients group is 1.87 is higher than the normal controls whose mean values of the PI of the left MCA is 0.95 (*p* < 0.05). While comparing the CRS-R scores, it has been observed that the flow velocity was not correlated with the state of the consciousness of the patients significantly in the level of *α* = 0.05. However, PI of the left MCA was significantly inversely correlated with the motor subscore of the tested patients whose CRS-R was in the level of *α* = 0.05 (Pearson’s product-moment correlation coefficient = −0.556, *p* = 0.025). Figure 3 shows a scatter diagram of PI of the left MCA and the motor subscores of CRS-R of the DOC patients.

**Table 2:** Left middle cerebral artery TCD results

	DOC			Controls			*p* (bilateral)
	Mean	SD	Range	Mean	SD	Range	
Systolic velocity	75.56	32.67	22–93	101.29	27.16	58–151	0.000
							0.000
End-diastolic velocity	23.31	21.92	0–42	41.19	15.89	23–59	0.000
							0.000
Mean velocity	38.88	23.14	7–59	62.90	16.28	38–84	0.000
							0.000
Pulsatility index	1.87	1.30	0.85–2.98	0.95	0.33	0.58–1.26	0.002
							0.004
Resistive index	0.76	0.16	0.54–1.00	0.59	0.11	0.44–1.00	0.002
							0.004

TCD results of the left middle cerebral artery comparing patients with normal subjects. Systolic velocity; mean velocity; the end-diastolic velocity was significantly reduced. Pulsatility index (PI) was significantly increased in all patients, as well as the resistive index

*SD* standard deviation

In HC2180-D, the PPG-derived parameters were calculated on the basis of digital volume pulse (DVP) similar to those for pressure wave. The Table 3 outlines PPG results of both the patients group and the healthy control sample. It is worth noting that the mean value of photoplethysmographic augmentation index (PAI) in DOC patient group was higher than the control group and the differences reached significance (*p* < 0.05). Figure 3 shows the normalized PPG signal and its feature point used in calculation of PAI for waveforms exhibiting. And in our study the PAI = (P2 − P1)/(P2 − P0). P2 = maximal point of DVP. P0 = minimal point of DVP.

**Table 3:** Peripheral vessel PPG results

	DOC			Controls			*p* (bilateral)
	Mean	SD	Range	Mean	SD	Range	
PAI	0.093	0.098	0.13–0.24	0.014	0.085	−0.13 to 0.22	0.014
							0.016

The Table 3 outlines PPG results of both the patients group and the healthy control sample. It is worth noting that the mean value of photoplethysmographic augmentation index (PAI) in DOC patient group was higher than the control group and the differences reached significance (*p* < 0.05)

P1 = inflection point that indicates the beginning up-stroke of the reflected wave [11].

**Fig. 4:**
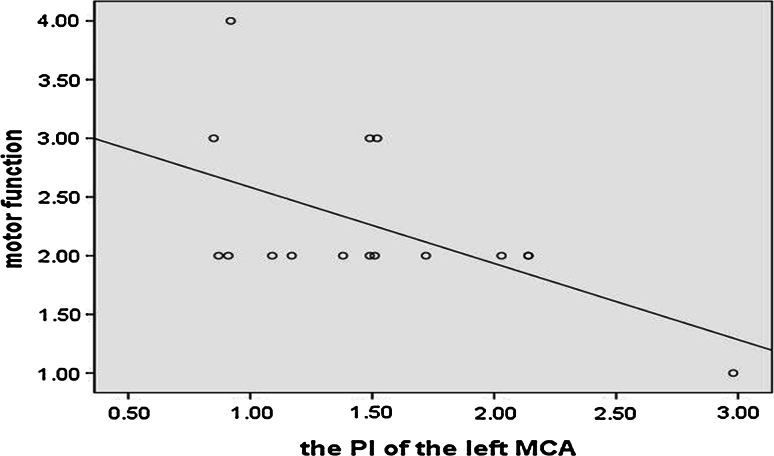
The scatter diagram of the PI of the left MCA and the motor subscores of the CRS-R of the DOC patients. In our research, we find that the PI of the MCA is inversely correlated with the motor subscore of our patients that include in their Coma Recovery Score-Revised (CRS-R) in the level of *α* = 0.05 (Pearson’s product-moment correlation coefficient = −0.556, *p* = 0.025)

No significant correlation between the PAI and the CRS-R has been observed here in the level of *α* = 0.05, but a medium level correlation between the PAI of PPG and the mean velocity of the left middle cerebral artery of TCD was found in all the groups in the level of *α* = 0.05 (Pearson’s product-moment correlation coefficient = −0.377, *p* = 0.022), as shown in Fig. 4—a scatter diagram of the PAI of PPG, and the mean velocity of the left middle cerebral artery.

## Discussion

**Fig. 5:**
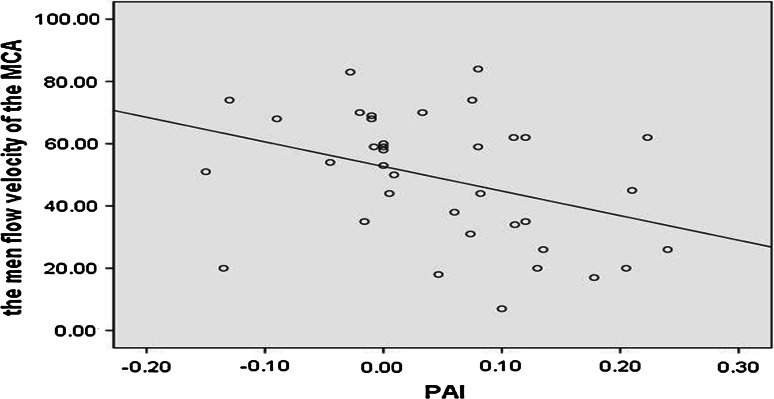
It is the scatter diagram of the PAI of the PPG and the mean velocity of left middle cerebral artery in all the groups. A medium level correlation between the PAI of PPG and the mean velocity of the left middle cerebral artery of TCD was found in all the groups in the level of *α* = 0.05 (Pearson’s product-moment correlation coefficient = − 0.377, *p* = 0.022)

According to the data, group differences between DOC patient and control in both the PAI of the PPG (photoplethysmographic augmentation index) and the PI of TCD (pulsatility index of the TCD) were confirmed in this study. As a result, the trend of the PI of TCD is consistent with the data shown in the literature [13]. The trend of PAI of PPG is first observed in our study. In comparison with the healthy control group, the DOC patients involved in the study displayed elevated values of PAI of PPG and the PI of TCD, with both differences reaching significance level (*α* = 0.05) (see Fig. 5).

Previous studies on the topic have already demonstrated cerebral flow and cerebral metabolism reduction of the entire brain in VS patients, measured with PET [14, 15]. Human and animal studies have proven a strong linkage between CBF and cerebral metabolic rate, as well as the fact that decreased cerebral metabolic demand in DOC patients leads to a decrease in their CBF [15–17]. The lowered flow velocities of the patients group used in this study, including systolic, end-diastolic, and mean velocities, indicates that CBF in DOC patients’ reduction is consistent with the earlier studies on the subject [18]. As shown by Gosling and King, PI is a reliable indicator when quantifying cerebral vascular resistance (CVR) [19]. Moreover, CVR increase has been observed to have positive correlation with PI [20, 21]. In this study, CVR level in DOC patients has been shown to exceed the normal.

Cerebral vascular resistance is dominated by intracranial pressure (ICP), blood viscosity and cerebral vascular caliber [22], with ICP of DOC patients being generally proven to stay within the normal range [13]. In this study, both the waveform of the TCD of DOC patients and their angiography were within normal, what is indicative of normal level of their cerebral vascular caliber.

Pulsatility index has been calculated by the velocity of the cerebral vascular, by subtracting end-diastolic velocities from systolic velocities, and then dividing the result by the mean velocities [PI = (*V*_s_ − *V*_d_)/*V*_m_]. All the parameters of TCD were compared and analyzed in all groups. This study has found that the flow velocities in the sample were inversely correlated to the PI of the TCD in the level of *α* = 0.05, with the only reasoning being that decreased flow velocities of cerebral vascular DOC patients can augment patients’ brain blood viscosity. Therefore, this disadvantageous factor can account for the elevated values of CVR in the DOC patients, which can lower the cerebral perfusion pressure—potentially being another adverse factor responsible for reducing CBF and cerebral metabolism. Furthermore, this study found that PI of the MCA is inversely correlated with the motor subscore of sample’s patients group that include in their CRS-R in the level of *α* = 0.05 (Pearson’s product-moment correlation coefficient = −0.556, *p* = 0.025). These results can possibly indicate that the changes of CVR in DOC patients may be accompanied with recovery of their neurological function. In addition, this research shows potential of using TCD (MCA PI) as an integral part of non-invasive routine clinical screening and assessment of DOC patients’ motor functions.

The increased PAI, which is an indicator of wave reflection, its trend was consistent with the results of augmentation index (AIx) obtained from the pulse pressure using applanation tonometry as an indicator of wave reflection within the arterial tree [11]. In some research, the PAI can measure the morphological changes by PPG signal. The increased PAI in our DOC patients suggest the shrink of their peripheral vascular which can augment peripheral vascular resistance and increase the afterload of their LV [23]. In this study, it has been proven that the value of the afterload of the cardiovascular system in DOC patients exceeds normal values. These changes can impair the function of the cardiac pump and by decreasing cardiac output (CO) reduce the CBF of the DOC patients, and affect the supply of oxygen and nutrients to the brain, decreasing cerebral metabolic rate. It has been proven that cerebral metabolic rate is closely linked to neurotransmitter recycling and restoration of neuronal membranes [24], therefore a lower cerebral metabolic rate in the DOC patients adversely affects their neural network recovery. With above in consideration, the PPG PAI is able to indirectly indicate the CO and cerebral metabolic rate and assist in more accurate assessment of their condition.

Despite the differences in the method of measurement and calculation, both the PI of TCD and the PAI of PPG can reflect the vascular resistance. Through analysis with Pearson’s product-moment correlation coefficient, an inverse correlation between the mean flow velocity of MCA and PPG PAI (*p* < 0.05) has been revealed. Further, due to inverse correlation of flow velocities with the PI of TCD (*p* < 0.05), the PAI of PPG and the PI of TCD exhibit an indirect relationship.

From a systemic standpoint of the DOC condition, in its initial stages patient’s neurological function recovery is associated with vital adaptations in the cardiovascular function; however, due to deconditioning of the DOC patients’ cardiovascular system, the sufficient supply of oxygen and nutrients to the brain becomes inhibited due to combination of significantly reduced cardiac output and shrunken vascular volumes [8]. In this research paper, we found that the deconditioning of cardiovascular system occurs with an associated and drastic increase in peripheral vascular resistance because the values of PAI in our patients are higher than the normal (*p* < 0.05). Moreover, we also found their effect on the cerebral vascular occurs with an associated and drastic increase in CVR due to the inverse correlation between the mean flow velocity of MCA and PPG PAI (*p* < 0.05) has been revealed in our research. Moreover, this study has also uncovered direct, or indirect correlation between the values of CVR and peripheral vascular resistance, and consciousness of the patients. Significant difference between the PI of TCD and the PAI of PPG value between DOC patients group and the healthy control group have been observed, hinting to a potential method of earlier prognosis of the DOC patients in clinic.

Despite hemodynamic changes of the cerebral vascular and the cardiovascular system in the DOC patients being essential in assessment of the state of their condition, few comparative studies utilizing them are been conducted. This study, utilizing both, has provided a clinical evidence of the existence of a direct and indirect link between deconditioning of the cerebral vascular and cardiovascular system of DOC patients, and their state of neurological function.

The inability to find the correlation between subscore of CRS-R and PI of the TCD, except for the motor subscore, has been seen as a limitation of this study. The linkage between PI and the motor subscore is relative easy to measure, due to blood supply of the precentral gyrus being dominated by the MCA. On the other hand, the other brain functional regions which can be assessed by the verbal, visual and communication subscores, possess relatively complex blood supplies due to their locations—that paired with technical restrictions of the TCD have it unfeasible to detect the deep distal cerebral vascular by TCD, making testing the correlation between PI and other subscores impossible.

Another constraint encountered while carrying out the research was the small sample of patients, which can be attributed to considerable rarity of patients with the studied condition. However, based on the significance of the results and their possible implications to patients’ well-being, it has been decided to report them.

In summary, this study presented an initial evaluation of the hemodynamic changes in relation to CVR and systemic vascular resistance, as well as their clinical performance. The results achieved revealed an indirect relationship between transcranial Doppler ultrasonography pulsatility index and finger photoplethysmography augmentation index in the patients in state of DOC. It has been established that quantitative comparison between data achieved through both of the above methods potentially enhances early prognosis capabilities in DOC patients in clinics.

## Conclusion

The results of this study revealed specific relationship between transcranial Doppler ultrasonography pulsatility index and finger photoplethysmography augmentation index in the DOC patients. That relationship can potentially be exploited to enhance the capabilities in early assessment of deconditioning of the patients’ cardiovascular system and its influence on their cerebral vascular system, ultimately predict tendency of the prognosis of the DOC patients in clinic.
